# Mixture cure model for estimating short-term and long-term colorectal cancer survival 

**Published:** 2019

**Authors:** Farzaneh Amanpour, Setareh Akbari, Mehdi Azizmohammad Looha, Mohammad Abdehagh, Mohamad Amin Pourhoseingholi

**Affiliations:** 1 *Gastroenterology and Liver Diseases Research Center, Research Institute for Gastroenterology and Liver Diseases, Shahid Beheshti University of Medical Sciences, Tehran, Iran*; 2 *Basic and Molecular Epidemiology of Gastrointestinal Disorders Research Center, Research Institute for Gastroenterology and Liver Diseases, Shahid Beheshti University of Medical Sciences, Tehran, Iran*; 3 *Department of Biostatistics, Faculty of Paramedical Sciences, Shahid Beheshti University of Medical Science, Tehran, Iran *

**Keywords:** Mixture cure model, Colorectal cancer, Survival

## Abstract

**Aim:**

We used mixture cure mode to separately investigate the risk factors for long-term and short-term survival of colorectal cancer patients.

**Background:**

Colorectal cancer (CRC) is the second most common cancer worldwide. In cancer studies, patients’ survival is the most important indicator of patients’ status. Classical methods in analyzing the survival data usually apply Cox proportional hazard regression.

**Methods:**

The study was performed on 1121 patients diagnosed with colorectal cancer. Mixture cure model with Weibull distribution and logit link function was fitted to data.

**Results:**

Odds of long-term survival for rectum cancer patients were lower than for colon cancer patients (OR=0.29(0.09, 0.9)). Also, patients with the advanced stage of the disease had lower odds of long-term survival compared to early-stage patients (OR=0.24(0.06, 0.86)).

In the short-term, the hazard of death for people with normal BMI was lower than the underweight group (HR=0.4(0.21, 0.76)). The short-term hazard of death for rectum cancer was about half of the short-term hazard for colon cancer (HR=0.49(0.29, 0.81)). Further, people with moderately (HR=2.11(1.26, 3.55)) and poorly (HR=4.04(2.03, 8.03)) differentiated tumor grade had a higher short-term hazard of death compared to people with well-differentiated grade.

**Conclusion:**

Predictive variables of colorectal cancer survival showed different effects in short- and long -terms. Site topography was a prognosis for both long-term and short-term survival; BMI and tumor grade were short-term predictors of survival while stage was a long-term predictor of survival.

## Introduction

 Colorectal cancer (CRC) is the second most common cancer worldwide, accounting for about 10.2% of total cancers. In Iran, it is the most prevalent cancer following breast cancer. In 2018, the estimated age-standardized incidence rates in Iran was 12.9 per 100000; the estimated number of 5-year prevalent cases was 24354 (31.9 per 100000) (1). Colorectal cancer has imposed a high burden on the Iranian society (2).

Several studies have investigated risk factors associated with colorectal cancer, including familial and hereditary factors as well as environmental lifestyle-related risk factors. Some risk factors related to colorectal risk and survival include sex, race and age, low physical activity, low fiber diet, obesity, smoking, diabetes, hypertension, alcohol consumption, diet high in fat especially from animal sources, diet low in fruits and vegetables, red meat, total energy intake, saturated fat, vitamin D, calcium, and aspirin (3-8).

In cancer studies, patients’ survival is the most important indicator of patients’ status. Classical methods in analyzing the survival data usually apply Cox proportional hazard regression. The popularity of the cox model is because it makes no assumption for the distribution of survival time. However, if the distribution of time is known, the traditional alternative to the Cox model is the parametric regression. The common distributions for survival time include Weibull, lognormal, log-logistic, and exponential. In short follow-up studies, these approaches assume that all patients experience the event of interest, and the hazard of the event is constant over time. However, in long follow-up studies, these assumptions may be violated, and other approaches should be considered (9). 

Progress in the treatment of cancer has led many patients to live a longer life. Thus, if the follow-up period is long enough, these patients may never experience the event of interest during the course of the study. As a result, the censoring rate increases, and the survival rate is overestimated.

The mixture Cure model can be an alternative to cox proportional hazard models in these situations. In such approaches, patients are divided into two groups of cured and non-cured, with each being modeled separately. Cured subjects are modeled through logistic regression, and their odds of long-term survival are investigated, while the uncured patients are modeled by a survival model, and their short-term hazard is calculated (10).

In this study, we fitted a parametric mixture model to colorectal cancer patients, and investigated the covariates affecting their short- term and long-term survival. 

## Methods

We performed the study on 1121 patients diagnosed with colorectal cancer who were referred to Taleghani Medical and Training Hospital, Tehran, Iran, between 2001 and 2007. The outcome was the survival time of patients which was defined as the time between diagnosis and patients’ death due to colorectal cancer. Patients who were alive at the end of the study were considered as right-censored. 

Variables in the study included age, sex, BMI, diabetes, hypertension, tumor grade, tumor size, stage, site topology, and family history. Variables were introduced initially into the crude mixture parametric cure model, and significant variables were fed into the multiple model.

The parametric mixture cure model with Weibull distribution and Logit link function was fitted to the data. The adequacy of the cure model was investigated via the Kaplan Mayer method. If the cure model is appropriate, Kaplan-Mayer graph shows a non-zero asymptote which indicates a large proportion of patients who will not experience the event.

The mixture cure model considers a population as a mixture of two groups: the cured and not cured. Consequently, the mixture cure model is a combination of two models.

Mixture cure survival model can be written as:

S (t) =πSU (t) +1-π 

Where, SU (t) is the survival function for uncured patients and π is the probability of not being cured.

The effect of covariates on π can be investigated by a logistic link:

Logit [π (z)] = z´γ

The effect of covariates on SU (t) can be modeled as follows: 

SU (t) =SU0 (t) exp (x´ß)

As a result, this model provided two separate interpretations of cured and uncured patients.

For cured patients, the effects of covariates on the cure probability are investigated though the logistic model, while for uncured patients, the effect of covariates on the distribution of time to event is investigated. In other words, the results of long-term survivors are interpreted by the odds ratio in the logistic model, while the results of short-term survivors are interpreted by hazard ratio in the parametric survival model. In this analysis, we used Weibull distribution, and we modeled the scale parameter and kept the shape parameter fixed so that the model would be considered as a proportional hazard model.

The analysis was performed by the CURETEG module in STATA15. 

## Results

The study was conducted on 1121 patients diagnosed with colorectal cancers. The mean age of patients at diagnosis was 53.56 (52.72, 54.40) years. The results of the descriptive statistics are shown in Table 1. The mean and median survival time was 105.08 (95%95.05, 115.12) and 95.43 (58.65, 130.42) months, respectively. A total of 235 (21%) patients died during the follow-up. The 1-, 3- and 5-year survival rates were 0.90(95%CI :(0.88, 0.92)), 0.73(95%CI :(0.70, 0.77)), and 0.61(95%CI: (0.55, 0.66)) respectively.

**Figure 1:**
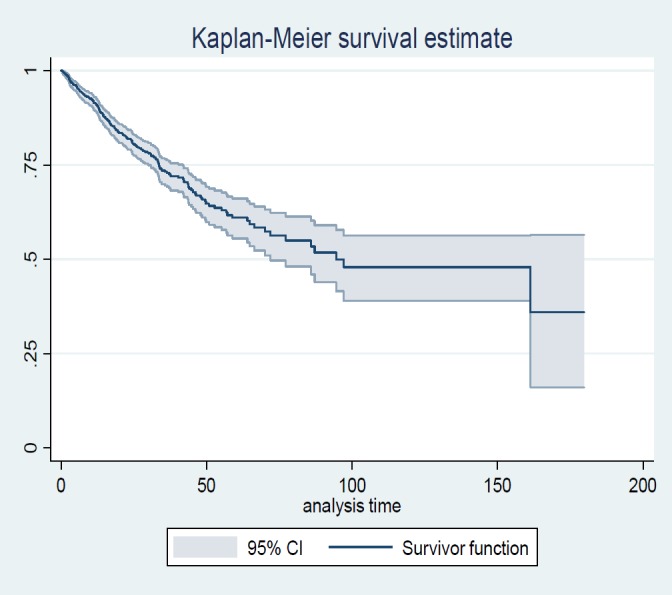
Overall survival curve with 95% CI for colorectal cancer patients

Kaplan Meier Survival curve with a 95% confidence interval is displayed in Figure 1. The figure indicates that the survival curve levels up at an approximate time of 100 months with the survival rate of about 50% associated with the patients who were considered long-term survivors. The results of the mixture cure model with Weibull distribution and Logistic link function are shown in Table 2. 

**Figure 2:**
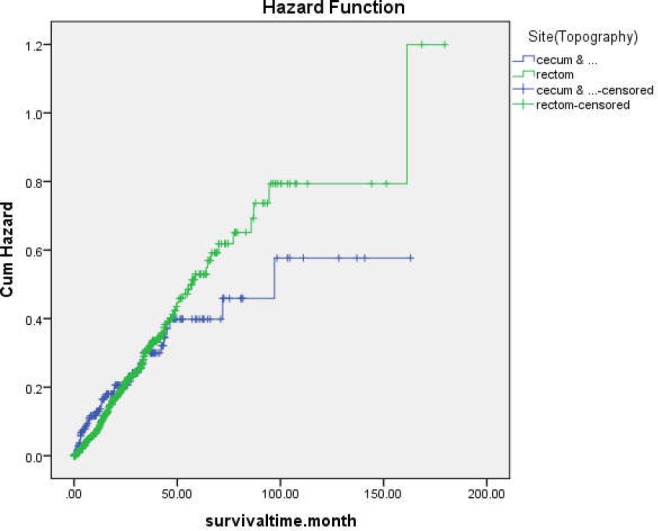
Kaplan-Meier curve for colorectal cancer hazard of death stratified by site

**Table 1:** Characteristics of colorectal cancer patients

variables	N%	Death%	Mean Survival Time (SE)	variables	N%	Death%	Mean survival time (SE)
Age				Tumor Grade			
<45	339(30.2)	73(21.5)	97.22(7.65)	Well differentiated	441(55.5)	66(15)	113.77(7.70)
45-65	511(45.6)	100(19.6)	117.49(6.51)	Moderately differentiated	284(35.7)	64(22.5)	74.46(3.85)
>65	271(24.2)	62(22.9)	79.108.58)	Poorly differentiated	70(8.8)	20(28.6)	71.05(6.67)
Sex				Tumor size			
Male	687(61.35)	149(21.7)	106.55(6.30)	<35	184(16.5)	30(16.3)	122.85(8.06)
Female	434(38.7)	86(19.8)	102.29(6.80)	>35	934(83.5)	205(21.9)	99.94(5.7)
BMI				Stage			
<18.5	65(9)	26(40)	64.80(12.1)	Early	437(45.2)	76(17.4)	118.76(7.13)
18.6-24.9	380(52.3)	79(20.8)	84.76(8.23)	advanced	529(54.8)	124(23.4)	90.48(7.19)
25-29.9	220(30.3)	27(12.3)	139.12(8.00)	Site topology			
>30	61(8.4)	8(13.1)	59.18(3.60)	Secum	234(21.2)	48(20.5)	108.09(7.92)
Diabetes				rectum	868(78.8)	183(21.1)	101.13(5.96)
Yes	77(8.7)	12(15.6)	77.85(7.33)	Family history			
No	805(91.3)	155(19.3)	105.70(6)	Yes	393(36.8)	80(20.4)	113.67(6.91)
Hypertension				No	675(63.2)	144(21.3)	94.26(6.43)
Yes	114(13.2)	19(16.7)	104.44(14.82)				
No	749(86.8)	145(19.4)	104.94(6.23)				

**Table 2:** Estimated odds ratio (OR) and hazard ratio (HR) by Weibull mixture cure model

Risk factor	Long term survival OR (95%CI)	p-value	Short term survival HR (95%CI)	p-value
Body Mass Index				
≤18.5	1		1	
18.6-24.9	0.41(0.07,2.32)	0.31	0.4(0.21,0.76)	0.01
25-29.9	3.48(0.65,18.65)	0.15	0.56(0.24,1.27)	0.16
≥30	3.79(0.51,28.18)	0.19	0.92(0.3,2.82)	0.88
Site Topography				
colon	1		1	
Rectum	0.29(0.09,0.9)	0.03	0.49(0.29,0.81)	0.01
pathologic stage				
Early	1		1	
				
advanced	0.24(0.06,0.86)	0.03	0.92(0.57,1.5)	0.74
Tumor Grade				
Well differentiated	1		1	
Moderately differentiated	1.85(0.45,7.6)	0.40	2.11(1.26,3.55)	0.01
Poorly differentiated	1.71(0.3,9.65)	0.55	4.04(2.03,8.03)	<0.01
Tumor size				
<35	1		1	
>35	1.1(0.27,4.53)	0.90	1.35(0.8,2.29)	0.27

The result showed that variables of site topography and the pathologic stage had a significant effect on long-term survival of patients. Odds of long-term survival for rectum cancer patients were lower than those of colon cancer patients (OR=0.29(0.09, 0.9)). Also, patients with the advanced stage of the disease had lower odds of long-term survival compared to early-stage patients (OR=0.24(0.06, 0.86)).

On the other hand, the variables of BMI, site topography, and grade had a significant effect on short-term survival of patients. The hazard of death for people with normal BMI was lower than that of the underweight group (HR=0.4(0.21, 0.76)). The hazard of death for rectum cancer was about half of the hazard for colon cancer (HR=0.49(0.29, 0.81)). Further, people with moderately (HR=2.11(1.26, 3.55)) and poorly (HR=4.04(2.03, 8.03)) differentiated tumor grade had a higher hazard of death compared to people with well-differentiated grade. The effect of site topography on hazard function is shown in Figure 2.

## Discussion

Variables in the study included age, sex, BMI, diabetes, hypertension, tumor grade, tumor size, stage, site topology, and family history. In our analysis, site topography had a significant effect on long-term as well as short-term survival of colorectal cancer patients. However, their effects were reversed. Odds of long-term survival for rectum cancer patients were lower than those of colon cancer patients; on the other hand, the short-term hazard of death for rectum cancer was less than the short-term hazard for colon cancer. This effect is shown in Figure 2. In classical survival analysis, this condition is considered a violation of proportional hazard assumption and the variable cannot be interpreted by hazard ratio; but the mixture cure model allows us to separate the effect of long-term and short-term survival and interpret them separately. Some studies discussed that cure model can be an alternative to cox semi-parametric model when the proportional hazard assumption is not satisfied (11).

 Age was not a significant factor in this study. Age was not significant either in similar studies conducted on this data (12). Some studies have shown that the patient's age is a predictor of survival for colon cancer, but not for rectal cancer (13). However, in some studies, older age was associated with a higher hazard of death in colorectal cancer patients (14-16). The discrepancy of our result with other studies may be due to distribution of age at diagnosis in Iran and western countries; in our study, the mean age at the time of diagnosis was 53.56 (SD=14.34) years which is somewhat equal to the reported age in Iranian studies (14, 17). However, the mean age of colorectal cancer patients in Iran is far lower than those in western countries (18, 19). Some studies indicated the increasing proportion of colorectal cancer in younger people (20). 

The variable of sex was not prognostic in this study. The results of some studies were in line with our study (12, 21); however, in some studies with a larger sample size, survival after colorectal cancer was longer in women than in men (22, 23).

The results of a meta-analysis showed that females had a higher overall survival (HR=0.85; 95% CI (0.85–0.89)) and higher cancer-specific survival (HR=0.92; 95% CI (0.89–0.95)) in colorectal cancer patients (24). Due to the low gender difference in colorectal cancer, a larger sample size may be required to show a significant effect.

In this study, BMI had a significant effect on short-term survival. Patients with normal BMI had a lower hazard of death compared to underweights. In a study with a semi-parametric model, the underweight BMI was associated with decreased cure probability (12). In other studies, higher BMI was associated with better survival (21, 25). However, some studies reported an association between a longer duration of overweight and increased risk of death in colorectal cancer patients (18). Diabetes and hypertension were not significant in this study. Some studies indicated diabetes as a prognostic factor for colorectal cancer (26). However, the association between diabetes and colorectal cancer is not conclusive as most studies did not account for all possible risk factors such as BMI and cancer stages in their study. Nevertheless, a review study concluded that diabetes is associated with lower survival of colorectal cancer patients (27). Another study showed no significant difference between the survival time of CRC patients suffering from hypertension and diabetes type 2 (28).

The variable of tumor grade had a significant effect on short-term survival of patients; however, it had no significant effect on long-term survival. In the short term, moderately and poorly differentiated tumors were associated with higher short-term hazard compared to well-differentiated tumors. Some studies obtained the same results (12, 16).

Variable tumor size was not significant in this study. One study indicated a positive correlation between tumor size and stage and grade, as well as an inverse association between tumor size and survival (29). The model in the present study was adjusted for stage and grade, and it was a possible reason for the non-significant effect of tumor size.

The variable stage had a significant effect on long-term survival of patients such that patients with advanced stages of the disease had lower odds of survival than patients in early stages. Other studies associated the early stage of cancer with better survival (14, 19).

In our analysis, site topography had a significant effect on long-term as well as short-term survival of colorectal cancer patients. However, their effects were reversed. Odds of long-term survival for rectum cancer patients were lower than colon cancer patients; on the other hand, the short-term hazard of death for rectum cancer was less than the short-term hazard for colon cancer. This effect is shown in Figure 2. In classical survival analysis, this condition is considered a violation of proportional hazard assumption and the variable cannot be interpreted by hazard ratio; but the mixture cure model allows us to separate the effect of long term and short-term survival and interpret them separately. Some studies discussed that the cure model can be an alternative to cox semi-parametric model when the proportional hazard assumption is not satisfied (11).

Family history was not significant in our study and other studies based on our data (21); one study indicated that having a family history was not associated with lower risk of death, but having two or more affected family members was associated with a lower risk of death (30); however, some studies found a significant association between survival of patients and family history of cancer (31).

The long-term survival can be modeled through the logit function while the short-term survival is modeled by parametric or semi-parametric distribution. We fitted the Weibull distribution for the short-term survival. Some studies showed that the Weibull model provides the best fit among parametric models to analyze the survival of colorectal cancer patients (21).

The results of this study are consistent with the results of other studies; however, there are some discrepancies. There were a few studies employing the mixture cure model to analyze the survival of colorectal cancer patients. 

In conclusion, predictive variables of colorectal cancer survival showed different effects in short- and long -terms. The mixture cure survival model provides separate investigation of the short-term and long-term survival rate, and gives better insight compared to standard survival models which only investigate the general survival of patients.
